# HNF4A Regulates the Formation of Hepatic Progenitor Cells from Human iPSC-Derived Endoderm by Facilitating Efficient Recruitment of RNA Pol II

**DOI:** 10.3390/genes10010021

**Published:** 2018-12-28

**Authors:** Ann DeLaForest, Francesca Di Furio, Ran Jing, Amy Ludwig-Kubinski, Kirk Twaroski, Amanda Urick, Kirthi Pulakanti, Sridhar Rao, Stephen A. Duncan

**Affiliations:** 1 Department of Cell Biology, Neurobiology and Anatomy, Medical College of Wisconsin, 8701 Watertown Plank Road, Milwaukee, WI 53226, USA; adelafor@mcw.edu (A.D.); ranjingpku@gmail.com (R.J.); aludwig@mcw.edu (A.L.-K.); ktwarosk@umn.edu (K.T.); aurick160@gmail.com (A.U.); 2 Department of Regenerative Medicine and Cell Biology, Medical University of South Carolina, Basic Science Building BS657A, 173 Ashley Ave, MSC 508, Charleston, SC 29425, USA; francescadifurio@gmail.com; 3 Blood Research Institute, BloodCenter of Wisconsin, Milwaukee, 8733 Watertown Plank Road, WI 53226, USA; Kirthi.Pulakanti@BCW.edu (K.P.); Sridhar.Rao@BCW.edu (S.R.)

**Keywords:** hepatocyte differentiation, transcription, liver development, induced pluripotent stem cells

## Abstract

Elucidating the molecular basis of cell differentiation will advance our understanding of organ development and disease. We have previously established a protocol that efficiently produces cells with hepatocyte characteristics from human induced pluripotent stem cells. We previously used this cell differentiation model to identify the transcription factor hepatocyte nuclear factor 4 α (HNF4A) as being essential during the transition of the endoderm to a hepatic fate. Here, we sought to define the molecular mechanisms through which HNF4A controls this process. By combining HNF4A chromatin immunoprecipitation (ChIP) followed by high-throughput DNA sequencing (ChIP-seq) analyses at the onset of hepatic progenitor cell formation with transcriptome data collected during early stages of differentiation, we identified genes whose expression is directly dependent upon HNF4A. By examining the dynamic changes that occur at the promoters of these HNF4A targets we reveal that HNF4A is essential for recruitment of RNA polymerase (RNA pol) II to genes that are characteristically expressed as the hepatic progenitors differentiate from the endoderm.

## 1. Introduction

Several transcription factors with roles in converting the endoderm to a hepatic fate have been identified (for reviews see [1,2,3]). For example, members of the FOXA and GATA families of pioneer transcription factors mark liver regulatory elements within the endoderm as competent to be expressed upon hepatic specification [4,5,6,7,8,9]. Several additional transcription factors, including TBX3, HHEX, PROX1 and HNF1B, control the onset of hepatic gene expression and expansion of the hepatic progenitor cells as the primary liver bud is formed [10,11,12,13].

Another liver enriched transcription factor, hepatocyte nuclear factor 4 α (HNF4A), is a member of the nuclear receptor family [14]. HNF4A has been shown to be a central regulator of hepatocyte differentiation and for maintaining liver functions in adult mice [15,16,17,18,19]. HNF4A also appears to be fundamental for establishing the network of transcription factors that governs the expression of hepatic mRNAs during mid-gestation stages of hepatogenesis [20]. Although HNF4A regulates gene expression in adult hepatocytes, loss of HNF4A has a more profound impact on the fetal liver [15,17,20]. The dependency on HNF4A during embryonic stages may be because transcriptional regulation of gene expression becomes stable as the network of liver transcription factors increases in complexity during cell maturation [17,21,22]. HNF4A is present at the onset of liver bud formation suggesting that it could facilitate specification of the hepatic progenitor cells in response to inductive cues [23,24]. Unfortunately, this hypothesis has been challenging to address using mouse models because HNF4A is essential for gastrulation, which precedes the onset of hepatic development. Nevertheless, when the defects to gastrulation are circumvented through the generation of tetraploid embryos, loss of HNF4A severely impacts the expression of liver mRNAs during formation of the liver bud [25].

Although mammalian genetic models of embryogenesis have been invaluable, such an approach has limitations. For example, the extent of molecular and biochemical analyses that can be performed on these models is limited by the amount of material. Fortunately, the directed differentiation of human pluripotent stem cells has emerged as an alternative model for the study of cell fate [26,27]. Several research groups have demonstrated that cells that share many characteristics with human hepatocytes can be generated from human embryonic (hESCs) and induced pluripotent (hiPSCs) stem cells [28,29,30,31,32,33]. This cellular model is particularly appealing because the differentiation process appears to recapitulate key stages that are known to occur during the formation of hepatocytes in vivo and is both efficient and reproducible [30,34]. In a recent study, we used HNF4A-depleted hESCs to address whether it was required to generate hepatic progenitor cells from definitive endoderm. As predicted, loss of HNF4A had a profound impact on the onset of hepatic gene expression, which we concluded was consistent with a role for HNF4A in converting the endoderm to a hepatic fate [35].

The requirement for HNF4A during hepatic progenitor cell (hepatoblast) formation is now well established; however, the molecular mechanism through which it regulates the transition of the endoderm to a hepatic fate and the onset of expression of hepatic genes is unclear. Transcription is dependent upon recruitment of RNA polymerase II (RNA pol II) to a pre-initiation complex that assembles at promoters prior to the onset of RNA synthesis. Initiation of transcription regulated by multiple proteins that result in the phosphorylation of the C-terminal domain of RNA pol II. RNA pol II promoter occupancy and initiation of RNA synthesis, can therefore be considered as separate events, either of which that could be influenced by HNF4A. HNF4A has been shown to associate with several coactivators including NCOA1 (also called SRC-1) [36], CITED2 [37], CBP/p300 [38,39], and PMRT1 [40]. The interaction of HNF4A with histone modifying enzymes could potentially facilitate recruitment of the RNA pol II pre-initiation complex (PIC). The finding that HNF4A directly interacts with TFIIB and the Mediator co-activator complex also supports such a mechanism [41,42]. In contrast, analyses of the human α-1 antitrypsin promoter have implied that regulation of gene expression by HNF4A may act independently of RNA pol II recruitment [43]. During the differentiation of human epithelial colorectal adenocarcinoma cells (Caco2), phosphorylated RNA pol II was found to be assembled within a pre-initiation complex prior to recruiting HNF4A [43]. In this study, the authors proposed that RNA pol II occupied the promoter prior to the onset of expression and that initiation of transcription resulted from recruitment of HNF4A and histone modifying enzymes [43]. It is, therefore, unclear whether HNF4a regulates the conversion of the endoderm to a hepatic fate primarily through facilitating recruitment of RNA pol II or by mechanisms that act subsequently. In the current study, we reveal that HNF4A primarily regulates the onset of hepatic gene expression during the endoderm to hepatic transition through recruitment of RNA pol II to the promoters of HNF4A target genes.

## 2. Materials and Methods

### 2.1. Cell Culture

Human K3 iPSCs [44] were maintained as described previously [45] using an IgG-Fc E-cadherin fusion protein [46] (StemAdhere, Primorigen Biosciences, Inc., Madison, WI, USA). The iPSCs were induced to differentiate toward a hepatic fate using modifications to the originally published protocol [30] that have been described in detail [45]. The generation and characterization of iPSCs in which HNF4A expression was depleted has also been described in detail [35].

### 2.2. Oligonucleotide Array Analysis

RNA was prepared using the RNeasy mini kit (QIAgen, Valencia, CA, USA). Total RNA (100 ng) was used to prepare cRNA that was hybridized to Primeview human gene expression arrays (Affymetrix, Santa Clara, CA, USA). Images were acquired using a GeneChip Scanner 3000 and primeview arrays were normalized and analyzed as we have described previously [47] except that data were processed using Partek Genomic Suite software (Partek, St. Louis, MO, USA). Gene ontology, functional annotation and upstream activator analyses were performed using both Ingenuity Pathways Analysis and DAVID. All genomic data is available through GEO as the following datasets: GSE104612, GSE104613, and GSE104779. 

### 2.3. Chromatin Immunoprecipitation and ChIP-Seq

Human iPSCs were induced to differentiate until the appropriate stage then fixed with 1% formaldehyde for 10 min. Fixation was stopped with 125 mM glycine for 5 min at room temp before cells were washed 2X with ice cold PBS. Cells were collected by centrifugation and re-suspended in PBS supplemented with protease inhibitor cocktail II (Calbiochem, San Diego, CA, USA). Cell pellets were finally collected by centrifugation before storage at −80 °C. To prepare for chromatin shearing, cells were lysed in cell lysis buffer (85 mM KCl, 5 mM PIPES, 0.5% NP40) by rotating for 10 min at 4 °C and nuclei were collected by centrifugation at 1700× *g* for 5 min at 4 °C. Nuclei were re-suspended and washed for 5 min ice cold PBS + protease inhibitor cocktail II before being collected by centrifugation and re-suspended in 1 mL of shearing buffer (10 mM TrisCl, 1 mM EDTA, 0.1% SDS pH 7.6 + 1 mM PMSF + protease inhibitor cocktail II). Nuclei were sheared for 15 min using a S220 Focused-ultrasonicator (Covaris, INC, Woburn, MA, USA) and insoluble material collected by centrifugation at 12,000× *g* at 4 °C. Chromatin was quantified using a protein assay (cat#500-0006, Biorad, Hercules, CA, USA) and 450 µg of chromatin was used for each chromatin immunoprecipitation (ChIP). After quantification, chromatin was diluted in ChIP dilution buffer (1.1% TritonX100, 0.01% SDS, 1.2 mM EDTA, 16.7 mM Tris-Cl pH8, 167 mM NaCl) and precipitated with antibody (HNF4A, 2 ug, sc-6556 and RNA pol II, 1 ug, sc-899; Santa Cruz Biotechnology, Santa Cruz, CA, USA). Complexes were collected using protein A+G magnetic beads (16–663, Millipore, Temecula, CA, USA), stringently washed then eluted using ChIP elution buffer (1% SDS, 0.5 M NaHCO3). Crosslinks were reversed with 3 M NaCl followed by treatment with proteinase K and RNase A. DNA was isolated by phenol chloroform extraction and ethanol precipitation and re-suspended in 50 uL of TE. Precipitated DNA was finally analyzed by quantitative PCR (qPCR) or by high throughput sequencing (BGI, Shenzhen, China). Raw sequence reads were aligned to the reference genome (NCBI 37/hg19) using BowTie 2 and Model Based Analysis of ChIP-Seq (MACS) was used for peak-calling with a two-sided and elevated *p*-value cut off (<10^−5^) to reduce identifying false-positive peaks. All genomic data is available through GEO as the following datasets: GSE104612, GSE104613, and GSE104779.

### 2.4. SYBR Green RT-qPCR

SYBR green real-time PCR (RT-PCR) was performed using RT2 SYBR Green Master mix from QIAgen. Quantitative RT-PCR was performed in 384-well plates using Biorad CFX-384 thermocycler. Standard curves were generated for each primer pair using serial dilutions of genomic DNA and accepted efficiency of primers was between 90% and 110%. The following primers (5′ to 3′) were used: APOA2: cattcccaggttcaaagcat, caccctcaggaatgttccac; SFRP5: gctgctggggaatcaaagat, agcacccactaagtattggct; N4BP2L1: cttggccaaagctgttgttt, gtccccagctttccaggtag; SLC35D1: tgttcggctttaactttggca, cgtggtggtgaataagagcg; APOB: gcatgtgagggtgaggaaat, gagtccagctgcagtgatga; ANKS4B: gttcagtgcctcgaaccttg, acttggccagatgtctctcc; PLA2G12B: agccacaaaaccaagaagcc, actccctaacctttgccctc; HNF4A: cccagaacaaggatccagaa, ccccaagtcaggcattctaa; F7: gcagcactgcagagatttca; ctgcccttccaccaagttta. 

### 2.5. Western Blotting

Protein from whole cell lysates was run on 4% to 12% Tris-Bis acrylamide gels (NP0321, Thermo Fisher Scientific, Waltham, MO, USA) using the NuPAGE system (Thermo Fisher Scientific, Waltham, MO, USA). Protein was transferred to PVDF blotting membranes (1620177, Bio-Rad, Hercules, CA 94547) using wet electroblot apparatus. Blots were blocked with 5% non-fat milk in TBST. Primary antibodies (HNF4A, 1:500, sc-6556; Santa Cruz Biotechnology, Santa Cruz, CA, USA and GAPDH, 1:1000, DB600-502; Novus Biologicals, Centennial, CO, USA) and secondary antibodies (Donkey anti-goat IgG HRP and Donkey anti-mouse IgG HRP) were applied to the blots in 5% non-fat milk in TBST. Three 5 min TBST washes were performed after each antibody incubation. Following the last wash, SuperSignal West Pico Chemiluminescent substrate (34080, Thermo Fisher Scientific) was applied to the blots for 3 min. The blots were then exposed to films (F-9023, GeneMate, VWR, Radnor, PA, USA), which were developed in an SRX-101A developer (Konica Minolta, Ramsey, NJ, USA).

## 3. Results

### 3.1. The Onset of HNF4A Expression Coincides with the Induction of Hepatic Gene Expression during the Differentiation of Human iPSCs

**Figure 1:**
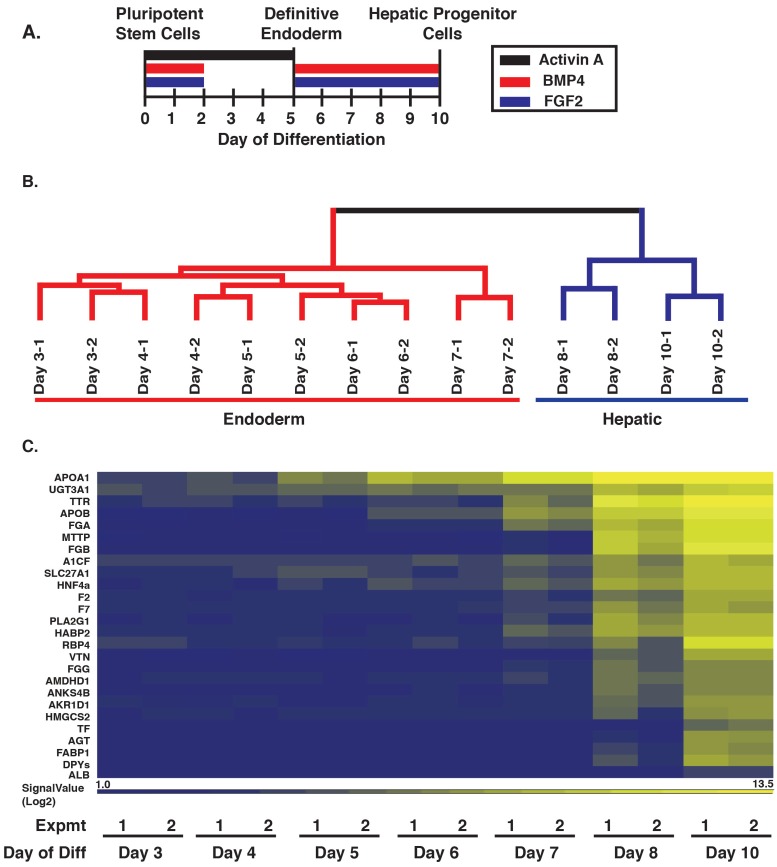
Transition of induced pluripotent stem cells (iPSC)-derived endoderm to a hepatic fate occurs between days seven and eight of differentiation. (**A**) Diagrammatic overview of the procedure used to generate hepatic progenitor cells from iPSCs that has been described in detail elsewhere [30,45]; (**B**) Dendrogram showing unsupervised hierarchical cluster analyses of transcriptional profiles generated using oligonucleotide arrays that were performed on duplicate differentiations collected daily between days three (endoderm) and day ten (hepatic progenitor cells). Note that cells with endoderm character cluster between days three and seven (red) and those with hepatic character cluster between days eight and ten (blue); (**C**) heat map showing the changes in levels of characteristic hepatic mRNAs during the differentiation time course.

We have previously described a protocol that generates cells with hepatocyte characteristics from human pluripotent stem cells [30,45,48]. The procedure relies on the generation of definitive endoderm by the addition of Activin A [49]. The endoderm is subsequently converted to a hepatic fate by the addition of BMP4 and FGF2 (Figure 1A). Our previous studies demonstrated that hepatic markers can be detected five days after the addition of BMP4/FGF2 to the endoderm [30,35]. Although expression of hepatic markers is indicative of the formation of the hepatic lineage, it was unclear exactly when the progenitors formed during this window of differentiation and at what stage expression of HNF4A was initiated. We, therefore, sought to discern the precise timing of hepatic progenitor cell specification in the iPSC differentiation model. 

RNA samples were collected daily between day three and day eight, as well as day ten, from two independent differentiations of wild-type iPSCs [1] (Figure 1A). Transcriptome analyses established the gene expression profile for each sample. Unsupervised hierarchical cluster analyses of the expression profiles demonstrated that the differentiating cells grouped into two distinct clades (Figure 1B). All samples collected between days three and seven clustered together suggesting that the cells all retained endoderm character. In contrast to the endoderm clade, samples from days eight and ten assembled as a distinct group, implying that the definitive endoderm adopted a new fate between days seven and eight of the differentiation protocol. We next examined the relative expression of 25 characteristic hepatic mRNAs using the same data sets. As illustrated by the heat map shown in Figure 1C, the majority of these hepatic mRNAs were undetectable during the first seven days of differentiation. By day eight, however, several hepatocyte mRNAs could be identified and the number of markers present increased through day ten of differentiation. Based on these studies of mRNA profiles we conclude that, under the conditions employed by our protocol, iPSC-derived definitive endoderm transitions to a hepatic fate between days seven and eight. 

**Figure 2:**
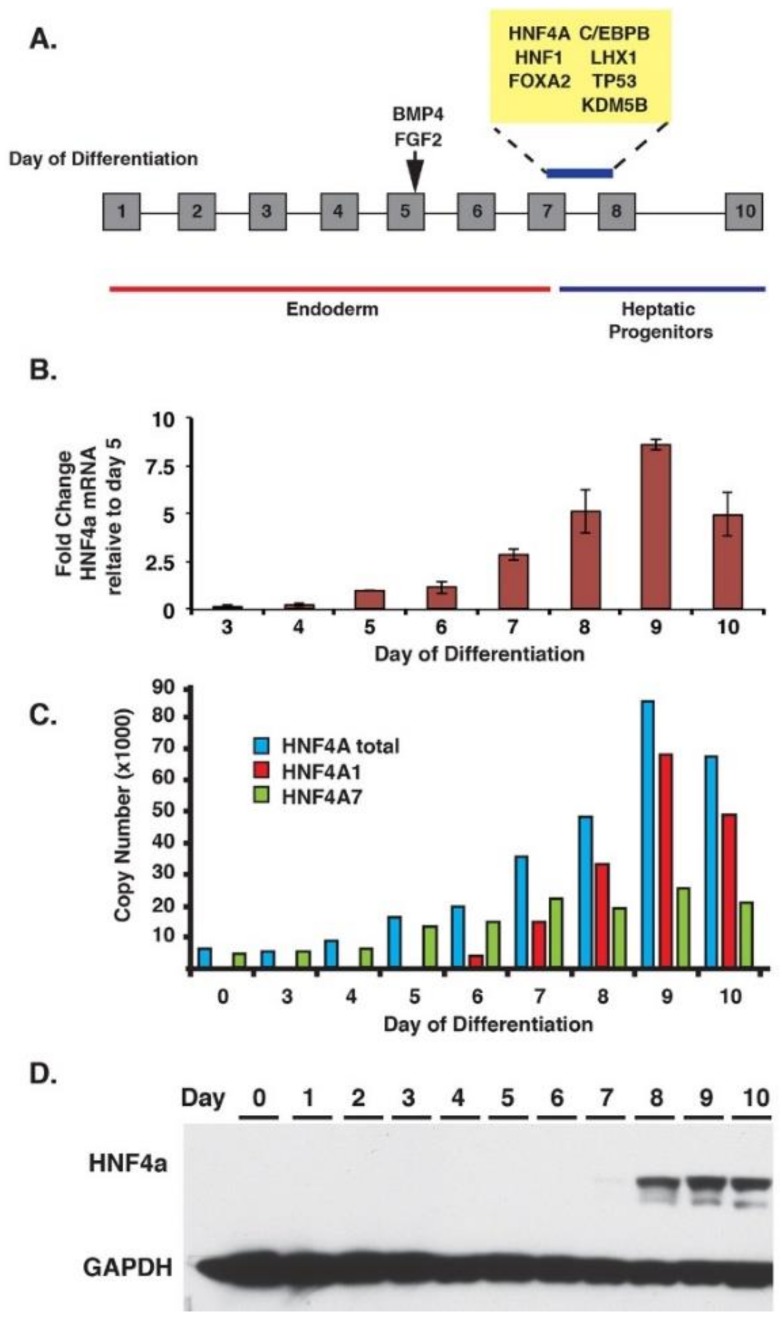
The onset of hepatocyte nuclear factor 4 α (HNF4A) expression coincides with the conversion of iPSC-derived endoderm to a hepatic fate. (**A**) Diagram summarizing the results of bioinformatics analyses of enriched transcription factor binding sites by Ingenuity Pathway Analyses. Several transcription factor binding sites (yellow box) were enriched in genomic sequences encoding proteins that are induced ≥4-fold at the endoderm (blue line) to hepatic (red line) transition, which occurs two days after addition of BMP4/FGF2 between days seven and eight of the differentiation process; (**B**) Bar graph showing relative levels of mRNA encoding HNF4A determined by real-time quantitative PCR (RT-qPCR) (standard error of the mean (s.e.m), *n* = 3 independent differentiations); (**C**) absolute levels of total *HNF4A* (blue), *HNF4A1* (red) and *HNF4A7* (green) mRNAs calculated by real-time RT-qPCR during the differentiation iPSCs to hepatic progenitor cells; (**D**) Immunoblot analyses of HNF4A protein levels throughout the differentiation time course.

We next sought to establish when HNF4A could first be detected compared to the onset of hepatic mRNA expression (Figure 2). We first used a bioinformatics approach to identify transcription factors that were potentially active at the start of hepatic progenitor cell formation. Based on the oligonucleotide array analyses, genes whose mRNA levels changed ≥4-fold between consecutive days of differentiation (*p*-value ≤ 0.01) were defined as being robustly induced. Analyses of the nucleotide sequence surrounding these genes by Ingenuity Pathway Analyses software (QIAGEN Redwood City 1001 Marshall Street, Suite 200 Redwood City CA 94063, United States) identified transcription factor binding sites that were over-represented in each gene set (Figure 2A). Within the gene set whose expression changed between days seven and eight, binding sites for HNF4A, HNF1, C/EBPB, and FOXA were all highly enriched. The identification of such binding sites is consistent with these transcription factors having known roles in regulating hepatic gene expression and suggested that they could act during the specification of the hepatic lineage [3]. We next established the timeline of HNF4A mRNA expression during the differentiation process. Real-time quantitative PCR (RT-qPCR) revealed the presence of trace levels of HNF4A mRNA by day five of differentiation; however, the levels increased substantially between days six and seven (Figure 2B). Several splice variants of HNF4A originate from two distinct RNAs called HNF4A1 and HNF4A7. The two HNF4A RNAs are regulated by independent promoters and, therefore, can be differentially expressed [50,51]. RT-qPCR using isoform-specific primers established the relative copy number of HNF4A1 and HNF4A7 mRNAs. Both promoters were active, but HNF4A1 accounted for the majority of HNF4A mRNAs induced at day seven of differentiation (Figure 2C). Since expression of mRNA precedes that of the encoded protein we next defined HNF4A protein levels by immunoblot (Figure 2D). HNF4A was detectable, albeit faintly, at day seven and the levels increased to around steady-state by day eight (Figure 2D). We conclude that expression of HNF4A, therefore, closely coincides with the onset of hepatic gene expression during the formation of hepatocyte progenitors from iPSC-derived endoderm.

### 3.2. Depletion of HNF4A Prevents Conversion of Definitive Endoderm to a Hepatic Fate

**Figure 3:**
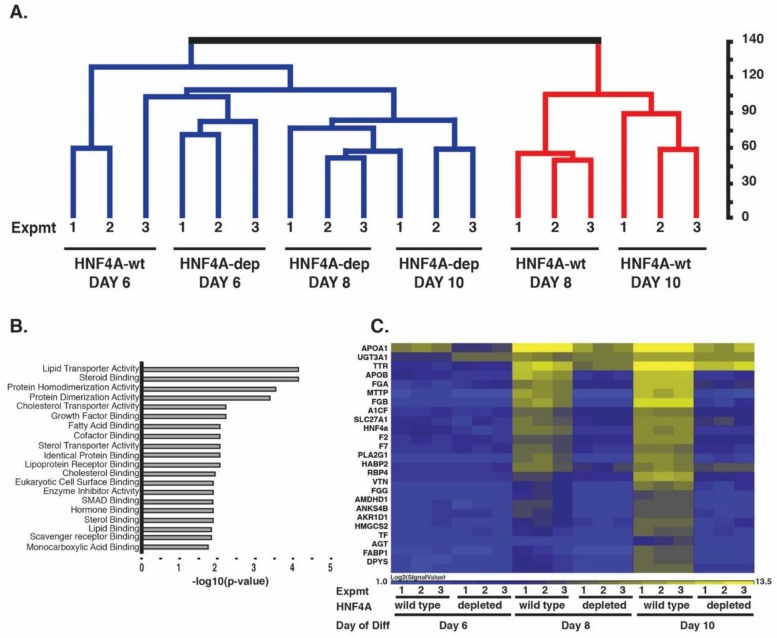
HNF4A is necessary for the transition of endoderm to hepatic fate during iPSC differentiation. (**A**) Dendrogram showing unsupervised hierarchical cluster analyses of transcriptional profiles generated using oligonucleotide arrays that were performed on triplicate differentiations of control and HNF4A-depleted cells collected immediately after addition of BMP4/FGF2 on day six (endoderm), day eight (early hepatic progenitor cells) and day ten (hepatic progenitor cells); (**B**) Gene ontology analyses of genes whose expression was reduced in the absence of HNF4A at day eight of differentiation; (**C**) Heat map showing the changes in levels of characteristic hepatic mRNAs at days six, eight, and ten of differentiation of control iPSCs (wild-type) and HNF4A-depleted (depleted) iPSCs. Scale = log2 of signal value.

In our previous studies, we reported that characteristic hepatic mRNAs are undetectable at day 10 of the differentiation protocol when expression of HNF4A is blocked [35]. While this finding was highly reproducible, the observation that HNF4A is first expressed between days seven and eight of differentiation raised the possibility that the reported phenotype at day ten reflects an indirect consequence of losing HNF4A. If HNF4A is indeed required to establish hepatocyte cell fate, we predicted that loss of HNF4A should prevent the initial expression of markers by day eight of differentiation. We tested this prediction by depleting HNF4A expression during iPSC differentiation using shRNAs as described previously [35]. Transcriptome analyses established the expression profiles in wild-type (iPSC K3) and HNF4A–depleted samples collected on day six (endoderm), day eight (onset of hepatic fate), and day ten (post-hepatic specification) of the differentiation procedure. Our previous studies had determined that hepatocyte differentiation was unaffected by a control shRNA and so we were confident that wild-type iPSCs were an adequate control line [35]. As expected, unsupervised hierarchical cluster analyses of the oligonucleotide array data revealed that wild-type day eight (onset of hepatic specification) and day ten (post-hepatic specification) profiles co-clustered. Moreover, this clade was distinct from wild-type cells at day six of differentiation (endoderm) (Figure 3A). HNF4A-depleted day six cells segregated with wild-type day six cells indicating that the presence of an HNF4A shRNA had little effect on endoderm formation. Importantly, when HNF4A-depleted cells were examined at days eight and ten, at which time wild-type cells had adopted a hepatocyte fate, the depleted cells co-clustered with day six wild-type (endoderm) cells. Gene ontology analyses revealed that transcripts reduced by the absence of HNF4A at day eight encoded proteins with roles commonly associated with hepatocyte function including steroid, lipid, and cholesterol metabolism (Figure 3B). Additionaly, the expression of mRNAs that are indicative of hepatocyte character were greatly inhibited in HNF4A–depleted cells compared to control cells at both day eight and day ten of differentiation (Figure 3C). This unbiased analysis demonstrates that during the differentiation of iPSCs toward a hepatic fate, loss of HNF4A prevents the formation of hepatic cells and the depleted cells instead retain endoderm characteristics.

### 3.3. HNF4A Occupies Regulatory Elements of Genes Expressed in Hepatocytes Coincident with Onset of Their Expression

**Figure 4:**
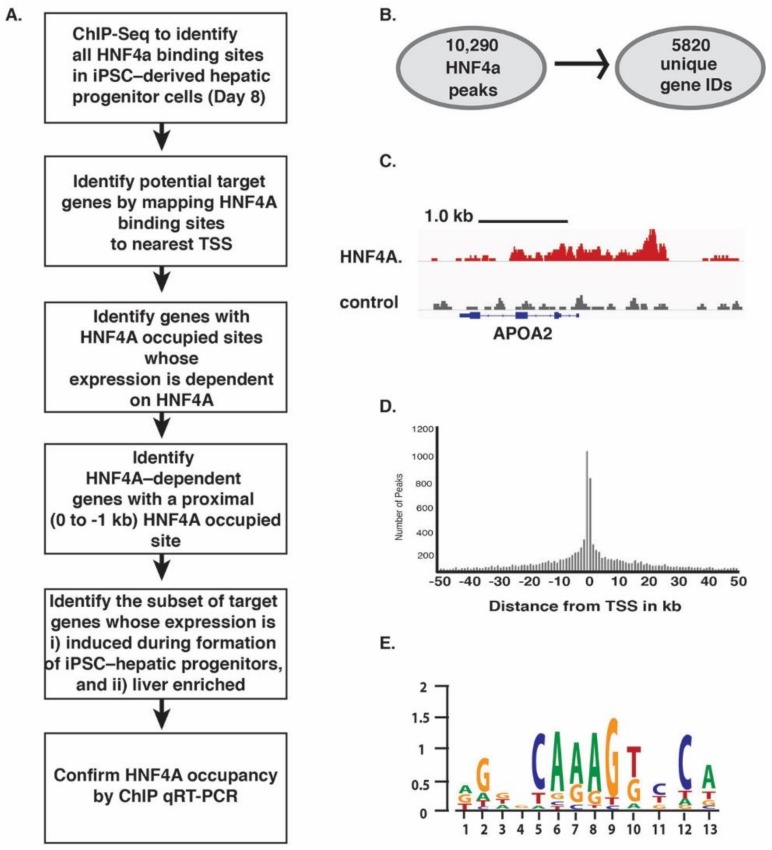
Identification of HNF4A bound sites in iPSC-derived hepatic progenitor cells. (**A**) Strategy used to identify direct targets of HNF4A during the formation of the hepatic lineage from iPSCs; (**B**) Summary of the number of targets identified by ChIP-seq analyses; (**C**) Example of HNF4A enriched sequences (peaks) in the *Apo2* gene; (**D**) Distribution of HNF4A peaks relative to the transcriptional start site of the nearest known gene; (**E**) Consensus HNF4A binding site determined from analyses of all peaks.

To establish how HNF4A controls hepatic progenitor cell formation required the identification of genes that were bound and regulated by HNF4A during the endoderm to hepatic transition (day eight) (Figure 4A). HNF4A occupied genomic sequences were identified at day eight of differentiation by ChIP followed by high-throughput DNA sequencing (ChIP-seq) [52]. Analysis of the ChIP-Seq data identified 10,290 peaks that mapped to 5820 unique genes (Figure 4B,C). The majority of HNF4A bound sites mapped within 10 kb of the predicted transcriptional start site of the gene (Figure 4D). DNA sequence analyses using Cistrome [53] revealed that the most highly represented motif within the sequenced fragments conformed to the published consensus HNF4A binding site sequence [21,54] (Figure 4E). 

**Figure 5:**
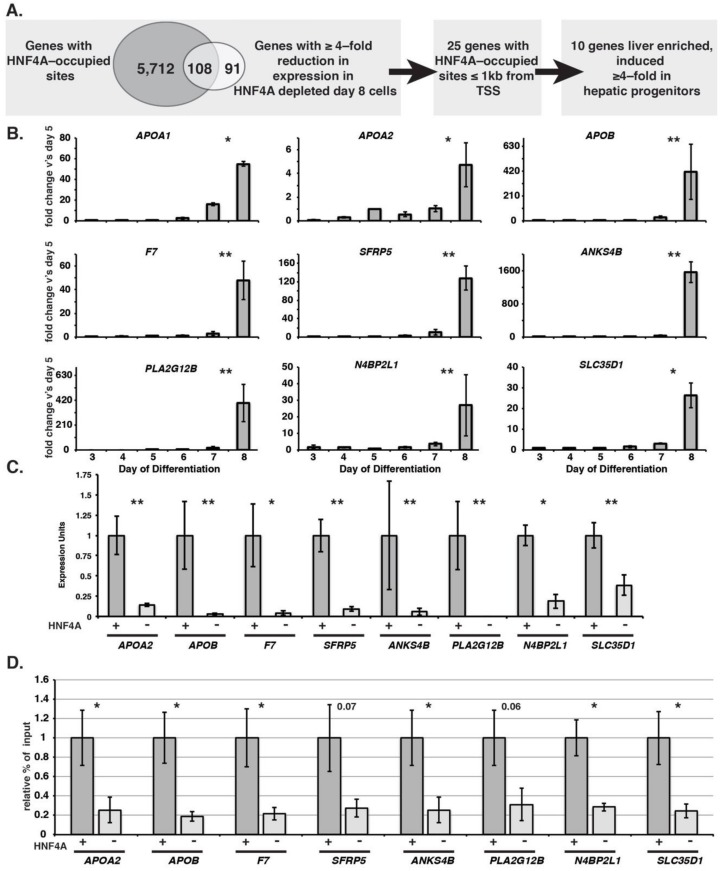
Identification of genes directly regulated by HNF4A during the endoderm to hepatic transition. (**A**) Diagram showing strategy used to identify genes that are direct targets of HNF4A with high confidence; (**B**) Expression profiles determined by RT-qPCR of genes that are direct targets of HNF4A during the transition of iPSC-derived endoderm to a hepatic fate. *t*-test compared expression levels at day eight vs. day seven; (**C**) Bar graphs showing RT-qPCR analyses of mRNAs expressed from HNF4A target genes in hepatic progenitor cells derived from control and HNF4A depleted iPSCs at day eight of differentiation. (**D**) Bar graphs showing results of ChIP-qPCR analyses of HNF4A occupancy in hepatic progenitor cells derived from control (dark grey) and HNF4A-depleted (light grey) iPSCs at day eight of differentiation. Error bars represent s.e.m, *n* = 3 independent differentiations. Student’s *t*-test, (*) *p* ≤ 0.05. (**) *p* ≤ 0.005.

We next sought to define a set of genes that were both associated with HNF4A occupied sites and whose expression was dependent upon HNF4A during the endoderm to hepatic transition (Figure 5A–D). We focused on identifying genes whose expression was reduced because HNF4A primarily acts as a transcriptional activator. The transcriptome data, described in Figure 3A, identified 199 genes whose expression was reduced by ≥4-fold in hepatic progenitor cells (day eight) after HNF4A was depleted (Figure 5A). A comparison of genes whose expression was dependent on HNF4A to those that had occupied HNF4A binding sites identified 108 genes that we concluded were direct targets of HNF4A. 

Further analyses focused on 25 genes that had an HNF4A-occupied site within 1 kb of the transcriptional start site. The proximity of these HNF4A bound sequences to the start-site implied that they contributed to a proximal promoter, which increased confidence that the site regulated expression of the target gene (Table 1). Gene expression profiles suggested that expression of a subset of the 25 HNF4A target genes occurred in a broad array of cell types or in the endoderm prior to HNF4A expression. Two more criteria were, therefore, placed on the candidate genes to identify those most likely to provide information that was relevant to the role of HNF4A in regulating onset of expression in hepatic progenitor cells. First, we retained 18 genes with enriched expression in the liver. Specifically, we used RNASeq data from Illumina’s Human BodyMap 2 that provides the level of mRNAs across 16 different tissues. We retained the genes whose expression was enriched in the liver greater than at least 13 other tissues in the panel (Table 1). Second, we retained only genes whose expression increased ≥4-fold between the endoderm (day five) and hepatic progenitor cells (day eight) generated from wild-type iPSCs (Figure 5B). Application of these rules left 10 genes, *APOA1, APOA2, APOB, F7, SFRP5, ANKS4B, PLA2G12B, N4BP2L1, SLC35D1,* and *HNF4A*, although we omitted HNF4A from the remaining analyses. Dependency on HNF4A for expression was confirmed for each of these genes by RT-qPCR on control and HNF4A-depleted cells at day eight of differentiation (Figure 5C). We next performed ChIP-qPCR to confirm whether HNF4A occupied the sites identified by ChIP-Seq (Figure 5D). With the exception of APOA1, for which we were unable to generate efficient qPCR primer pairs that flanked the HNF4A binding site, we were able to confirm occupancy by HNF4A of all the predicted binding sites. We conclude that we have identified eight representative genes whose expression is robustly induced during the endoderm to hepatic transition and is dependent on direct regulation by HNF4A.

### 3.4. HNF4A is Necessary for Recruitment of RNA pol II to Promoters of Target Genes

**Figure 6:**
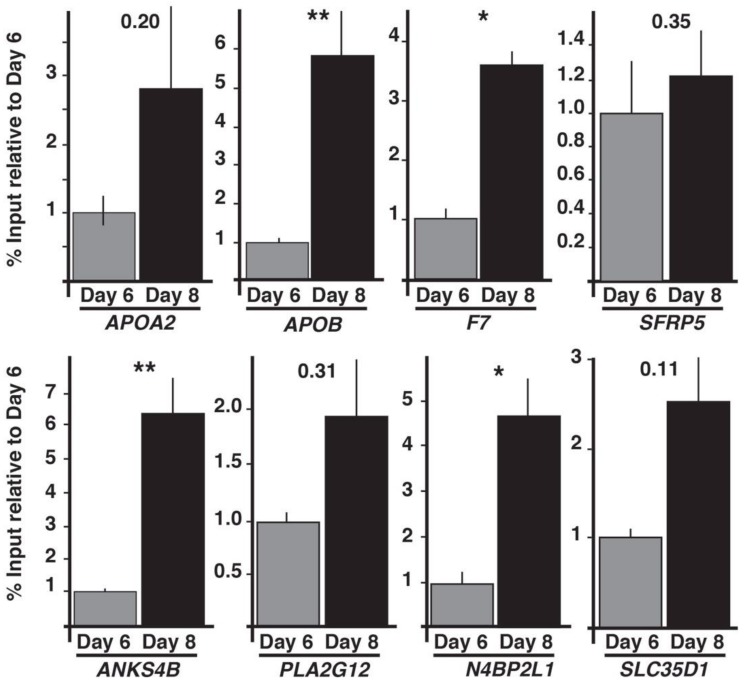
RNA polymerase II (RNA pol II) is recruited to HNF4A target genes coincident with the onset of expression. Bar graphs showing the results of ChIP-qPCR analysis of RNA pol II occupancy of HNF4A target genes during the transition of the endoderm (day six, grey bars) to a hepatic fate (day eight, black bars). For all graphs error bars = s.e.m., *n* = 3 independent differentiations. Student’s *t*-test, (*) *p* ≤ 0.05. (**) *p* ≤ 0.005.

**Figure 7:**
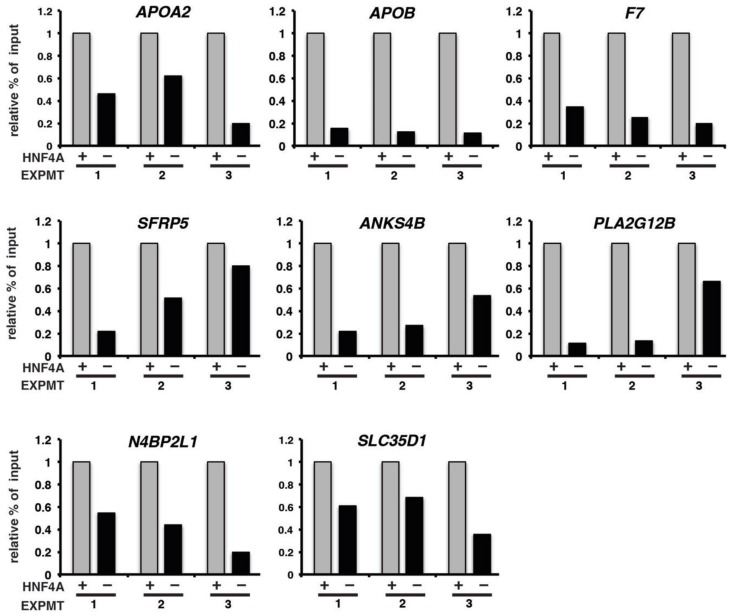
Recruitment of RNA pol II is decreased at HNF4A target genes when HNF4A is depleted. Bar graph showing the results of ChIP-qPCR analysis of RNA pol II occupancy of HNF4A target genes at the onset of HNF4A expression (day 8) in control and HNF4A-depleted iPSCs.

With high confidence HNF4A targets identified we first determined whether RNA pol II was pre-assembled at the promoters of the HNF4A target genes prior to their expression or whether RNA pol II was recruited to promoters as the endoderm transitioned to a hepatic fate at day eight. ChIP-qPCR was performed to detect RNA pol II at the promoters of HNF4A target genes in wild-type cells collected at day six (pre-HNF4A; pre-hepatic endoderm) or day eight (post-HNF4A; hepatic progenitor cells) (Figure 6). With the exception of *SFRP5* that had similar levels of RNA pol II occupancy at both day six and day eight of differentiation, the level of RNA pol II occupancy detected in the day six (pre-HNF4A) cells was minimal at all promoters. However, by day eight, when HNF4A expression is robust and expression of target genes has been initiated, RNA pol II occupancy increased substantially. Since RNA pol II occupancy coincided with expression of HNF4A, we next examined whether HNF4A was required for recruitment of RNA pol II to the promoters of HNF4A target genes (Figure 7). Control and HNF4A–depleted cells were induced to differentiate to day eight, when control cells form hepatic progenitors and HNF4A is normally expressed, and the extent of RNA pol II occupancy of HNF4A targets was measured by ChIP-qPCR. As before, RNA pol II was present at the promoters of all genes in control cells. In contrast, although there was some quantitative variability between differentiations, in cells in which HNF4A was depleted, all of the genes examined revealed a substantial reduction in RNA pol II occupancy (Figure 7).

Based on these cumulative data we conclude that the primary mechanism through which HNF4A converts the endoderm to a hepatic fate is by recruitment of RNA pol II to promoters of genes that are characteristically expressed during the formation of hepatocyte progenitors. 

## 4. Discussion

### 4.1. The Onset of Expression of Several Genes Enriched in Hepatocytes Requires HNF4A

We previously exploited the ability to differentiate human iPSCs into hepatic progenitor cells to reveal that that nuclear receptor HNF4A is required for hepatic progenitor cell formation (DeLaForest et al. [35]). In the current study, we have addressed the molecular mechanism through which HNF4A mediates this conversion. By dividing the differentiation procedure into 24-h intervals and examining the changes in gene expression profiles, we defined the timeframe through which iPSC-derived endoderm converts to a hepatic fate. The onset of expression of hepatic mRNAs initiates around 48–72 h after the addition of inductive growth factors (days seven to eight). We also mapped the onset of HNF4A expression and revealed that it coincides with this endoderm to hepatic transition. Moreover, when HNF4A is depleted, the endoderm fails to adopt a hepatic fate, but instead retains endoderm character. The 72-h lag between addition of inductive signaling molecules and the detection of HNF4A mRNA would suggest that the HNF4A gene is likely indirectly dependent on BMP/FGF. Moreover, this substantial delay in HNF4A expression would imply the existence of unappreciated regulatory pathways that occur between induction and fate conversation. The relationship between BMP signaling and induction of hepatic fate is currently under study. However, we have recently defined the repertoire of genes whose expression in the endoderm is directly regulated by FGF [55]. FGF immediate early response genes include those encoding transcription factors, modulators of cell signaling and epigenetic regulators. In addition to proteins that regulate transcription of the *HNF4A* gene, we have recently used a small molecule screen to reveal that regulation of HNF4A protein levels also contribute to the formation of hepatocytes from iPSCs [56]. It therefore seems reasonable to propose that several proteins coordinate to control the cellular environment during hepatocyte progenitor cell formation that ultimately regulates expression of transcriptional effectors including HNF4A. 

### 4.2. HNF4a Directly Binds Genes Whose Expression in Hepatic Progenitors is HNF4A-Dependent

In order to determine the mechanism through which HNF4A controls the formation of hepatic progenitor cells required us to identify genes that were direct targets of HNF4A transcriptional activity during the endoderm to hepatic transition. Although a number of genome wide studies have identified HNF4A binding sites in human hepatocytes and hepatoma cells [21,22,57,58,59], binding during the formation of the hepatic progenitor cells has to our knowledge not been described. Our lack of understanding of HNF4A occupancy during the endoderm to hepatic transition reflects the difficulty in obtaining enough embryonic material that is compatible with ChIP-Seq approaches. Moreover, ChIP-Seq analyses of evolutionarily distinct species have revealed that binding site location in general is species specific, meaning only a limited understanding of binding site location can be obtained from non-human embryos [59]. Fortunately, the availability of a human pluripotent stem cell differentiation model that recapitulates key aspects of hepatocyte differentiation, is synchronous, and reproducible, circumvents these challenges [30,34]. Using ChIP-Seq we identified 10,290 peaks that represent binding sites that become occupied by HNF4A during the formation of the hepatic progenitors. ChIP-Seq analyses of adult human liver samples have previously revealed the presence of 20,115 HNF4A occupied sites [59]. The fact that adult livers have more occupied sites than progenitor cells is unsurprising and likely reflects the increase in gene expression complexity that occurs as the hepatic progenitor cells mature to adult hepatocytes [20]. The observation that there exists a subset of sites in iPSC-derived hepatic progenitors that are not occupied in mature livers is more difficult to explain. The divergence in the data sets could reflect technical differences between experimental approaches. However, ChIP-qPCR revealed an enrichment of HNF4A occupancy in predicted binding sites of eight selected genes in control hepatic progenitor cells compared to HNF4A-depleted cells suggesting that our ChIP-Seq analysis was effective. Another explanation for divergence between the data sets is the possibility that there exist inherent differences in gene regulation between iPSC-derived cells and endogenous tissues. However, the explanation that we currently favor is that transcriptional regulation during cell differentiation is dynamic. Consequently, as cells mature the binding sites used to control transcription of a given gene can alter as the repertoire of transcription factors within the cell changes. Experiments to distinguish such possibilities are currently underway. 

The 10,290 HNF4A bound sequences mapped to 5820 unique genes. Having identified the genomic profile of HNF4A bound sequences in the hepatic progenitor cells we next determined whether expression of genes associated with the bound sites was dependent on HNF4A. Transcriptome analyses had demonstrated that expression of 199 genes was reduced by ≥4-fold in hepatic progenitor cells (day eight) after HNF4A was depleted. Of these genes, 54% (108 genes) had HNF4A occupied sequences. We conclude that many of these genes are directly regulated by HNF4A and that HNF4A mediates differentiation of the endoderm by controlling the expression of genes that are characteristic of the hepatic lineage. However, we recognize that HNF4A occupancy was undetectable in 46% of genes whose expression is lost during the differentiation of HNF4A–depleted cells. HNF4A occupancy of some of these genes may have been missed due to the technical parameters used to analyze the ChIP-Seq data. However, it is also probable that expression of these genes is affected as a secondary consequence or is regulated by the action of HNF4A at far distal enhancers. 

### 4.3. Depletion of HNF4a Reduces RNA pol II Recruitment to Promoters of HNF4A Target Genes

Studies of transcriptional regulation of α-1-antitrypsin (*SERPINA1*) during the differentiation of Caco2 cells, revealed that a complete pre-initiation complex that included RNA pol II was pre-assembled long before the onset of expression and recruitment of HNF4A [43]. In contrast to assembly of the pre-initiation complex, recruitment of SMARCA2 (hBRM) and HNF4A was coincident with transcriptional initiation. These data imply that the mechanism through which HNF4A controls expression is independent of recruitment of RNA pol II. However, Caco2 is an epithelial cell line isolated from a colorectal adenocarcinoma and so it was unclear whether the action of HNF4A would be the same during the formation of hepatic progenitor cells. This is especially true given that the endoderm converts to a hepatic fate occurs over 72 h of culture, whereas differentiation of Caco2 cells from a crypt-like to villus-like phenotype takes approximately 1 week [43]. 

Our examination of the presumptive promoters of eight genes occupied by HNF4a revealed that RNA pol II was recruited coincident with the onset of their expression. However, one gene, *SFRP5*, was an exception in that the level of RNA pol II identified at the promoter was similar in both the pre-hepatic and hepatic progenitor cell samples. When we examined the impact of depletion of HNF4a on the presence of RNA pol II, we found that the level of RNA pol II was reduced in the majority (7/8) genes examined. Based on these results we conclude that HNF4a mediates the onset of hepatic gene expression primarily by facilitating the recruitment of RNA pol II. We do not know whether HNF4a is physically involved in recruiting the polymerase complex or whether it does so indirectly. HNF4a interacts with TFIIB and the Mediator co-activator complex which may imply that HNF4a could directly recruit or at least stabilize the interaction of the basal transcription factor complex with target promoters [41,42]. However, HNF4A has also been shown to associate with a number of coactivators including SRC-1 [36], CITED2 [37], CBP/p300 [38,39], and PMRT1 [40]. Such interactions could potentially impact RNA pol II occupancy of promoters through mediating histone modification and nucleosome positioning. Of course, not all regulatory elements will necessarily respond to HNF4a occupancy identically. The response of HNF4a binding will likely be influenced by the specific co-activators and the repertoire of proteins present at a given regulatory element. With this in mind, it will most likely be useful to examine the impact of loss of HNF4a on histone modifications on a genomic scale. 

## Figures and Tables

**Table 1:** Direct targets of HNF4A expressed during the endoderm to hepatic transition. N.D: not determined.

GENE	EXPRESSION CHANGE IN DAY 8 HNF4A-DEPLETED CELLS (*p*-Value < 0.05, Array Data)	HNF4A BINDING SITE POSITION FROM TSS	CHANGE IN EXPRESSION IN HNF4A-DEPLETED HEPATIC PROGENITORS (qRT-PCR)	EXPRESSION ENDODERM V’S HEPATIC PROGENITORS BY qRT-PCR	EXPRESSION ENRICHED IN LIVER OVER 80% OF OTHER TISSUES	HNF4A OCCUPANCY (ChIP)
***SLC22A23***	−4.25777	−978	N.D	N.D.	yes	N.D
*APOA2*	**−7.00843**	**−838**	**−6.98**	**4.74**	**yes**	**yes**
*SFRP5*	**−5.56874**	**−789**	**−10.9**	**128.02**	**yes**	**yes**
***AKAP1***	−5.1654	−714	N.D	N.D.	no	N.D
***APOA1***	−11.2129	−319	−13.42	54.92	yes	N.D
***PHACTR2***	−4.99637	−250	−1.46	0.91	no	N.D
***C3orf15***	−4.33119	−249	−6.6	23.47	no	N.D
***EPHX2***	−4.47046	−238	−2.67	7.57	yes	no
*N4BP2L1*	**−8.12692**	**−230**	**−5.86**	**26.96**	**yes**	**yes**
***CHST13***	−4.49952	−184	N.D	N.D.	yes	N.D
*SLC35D1*	**−4.73481**	**−176**	**−2.67**	**26.33**	**yes**	**yes**
***VTN***	−7.51883	−163	−3.97	23.04	yes	no
***APOM***	−8.64496	−158	−2.22	1.51	yes	no
*APOB*	**−74.4626**	**−156**	**−30.75**	**31.69**	**yes**	**yes**
***EFEMP1***	−5.90295	−147	−4.53	23.08	no	N.D
***GLYCTK***	−6.10731	−140	−7.18	8.99	yes	N.D
*ANKS4B*	**−10.9033**	**−131**	**−15.46**	**351.45**	**yes**	**yes**
***LRP2***	−14.7087	−131	−18.87	152.12	no	N.D
*PLA2G12B*	**−30.8129**	**−114**	**−994.4**	**397.18**	**yes**	**yes**
***LGALS2***	−45.8533	−102	−75.65	19.83	no	N.D
***AQP11***	−5.36658	−95	N.D	N.D.	yes	N.D
***AGMAT***	−5.7087	−90	N.D	N.D.	yes	N.D
*HNF4A*	**−27.3065**	**−82**	**-**	**5.1**	**yes**	**yes**
***GDF15***	−6.02312	−74	N.D	N.D.	no	N.D
*F7*	**−8.84201**	**−71**	**−30.51**	**48.04**	**yes**	**yes**

## References

[1] Si-Tayeb K., Lemaigre F.P., Duncan S.A. (2010). Organogenesis and development of the liver. Dev. Cell.

[2] Zaret K.S., Carroll J.S. (2011). Pioneer transcription factors: Establishing competence for gene expression. Genes Dev..

[3] Lemaigre F.P. (2009). Mechanisms of liver development: Concepts for understanding liver disorders and design of novel therapies. Gastroenterology.

[4] Cirillo L.A., Lin F.R., Cuesta I., Friedman D., Jarnik M., Zaret K.S. (2002). Opening of compacted chromatin by early developmental transcription factors HNF3 (FoxA) and GATA-4. Mol. Cell.

[5] Lee C.S., Friedman J.R., Fulmer J.T., Kaestner K.H. (2005). The initiation of liver development is dependent on Foxa transcription factors. Nature.

[6] Bossard P., Zaret K.S. (1998). GATA transcription factors as potentiators of gut endoderm differentiation. Development.

[7] Cirillo L.A., McPherson C.E., Bossard P., Stevens K., Cherian S., Shim E.Y., Clark K.L., Burley S.K., Zaret K.S. (1998). Binding of the winged-helix transcription factor HNF3 to a linker histone site on the nucleosome. EMBO J..

[8] Taube J.H., Allton K., Duncan S.A., Shen L., Barton M.C. (2010). FOXA1 functions as a pioneer transcription factor at transposable elements to activate AFP during differentiation of embryonic stem cells. J. Biol. Chem..

[9] Sodhi C.P., Li J., Duncan S.A. (2006). Generation of mice harbouring a conditional loss-of-function allele of Gata6. BMC Dev. Biol..

[10] Seth A., Ye J., Yu N., Guez F., Bedford D.C., Neale G.A., Cordi S., Brindle P.K., Lemaigre F.P., Kaestner K.H. (2014). Prox1 ablation in hepatic progenitors causes defective hepatocyte specification and increases biliary cell commitment. Development.

[11] Lokmane L., Haumaitre C., Garcia-Villalba P., Anselme I., Schneider-Maunoury S., Cereghini S. (2008). Crucial role of vHNF1 in vertebrate hepatic specification. Development.

[12] Suzuki A., Sekiya S., Buscher D., Izpisua Belmonte J.C., Taniguchi H. (2008). Tbx3 controls the fate of hepatic progenitor cells in liver development by suppressing p19ARF expression. Development.

[13] Ludtke T.H., Christoffels V.M., Petry M., Kispert A. (2009). Tbx3 promotes liver bud expansion during mouse development by suppression of cholangiocyte differentiation. Hepatology.

[14] Sladek F.M., Zhong W.M., Lai E., Darnell J.E. (1990). Liver-enriched transcription factor HNF-4 is a novel member of the steroid hormone receptor superfamily. Genes Dev..

[15] Parviz F., Matullo C., Garrison W.D., Savatski L., Adamson J.W., Ning G., Kaestner K.H., Rossi J.M., Zaret K.S., Duncan S.A. (2003). Hepatocyte nuclear factor 4α controls the development of a hepatic epithelium and liver morphogenesis. Nat. Genet..

[16] Hayhurst G.P., Lee Y.H., Lambert G., Ward J.M., Gonzalez F.J. (2001). Hepatocyte nuclear factor 4α (nuclear receptor 2A1) is essential for maintenance of hepatic gene expression and lipid homeostasis. Mol. Cell Biol..

[17] Battle M.A., Konopka G., Parviz F., Gaggl A.L., Yang C., Sladek F.M., Duncan S.A. (2006). Hepatocyte nuclear factor 4α orchestrates expression of cell adhesion proteins during the epithelial transformation of the developing liver. Proc. Natl. Acad. Sci. USA.

[18] Watt A.J., Garrison W.D., Duncan S.A. (2003). HNF4: A central regulator of hepatocyte differentiation and function. Hepatology.

[19] Garrison W.D., Battle M.A., Yang C., Kaestner K.H., Sladek F.M., Duncan S.A. (2006). Hepatocyte nuclear factor 4α is essential for embryonic development of the mouse colon. Gastroenterology.

[20] Kyrmizi I., Hatzis P., Katrakili N., Tronche F., Gonzalez F.J., Talianidis I. (2006). Plasticity and expanding complexity of the hepatic transcription factor network during liver development. Genes Dev..

[21] Bolotin E., Liao H., Ta T.C., Yang C., Hwang-Verslues W., Evans J.R., Jiang T., Sladek F.M. (2010). Integrated approach for the identification of human hepatocyte nuclear factor 4α target genes using protein binding microarrays. Hepatology.

[22] Odom D.T., Zizlsperger N., Gordon D.B., Bell G.W., Rinaldi N.J., Murray H.L., Volkert T.L., Schreiber J., Rolfe P.A., Gifford D.K. (2004). Control of pancreas and liver gene expression by HNF transcription factors. Science.

[23] Duncan S.A., Manova K., Chen W.S., Hoodless P., Weinstein D.C., Bachvarova R.F., Darnell J.E. (1994). Expression of transcription factor HNF-4 in the extraembryonic endoderm, gut, and nephrogenic tissue of the developing mouse embryo: HNF-4 is a marker for primary endoderm in the implanting blastocyst. Proc. Natl. Acad. Sci. USA.

[24] Taraviras S., Monaghan A.P., Schutz G., Kelsey G. (1994). Characterization of the mouse *HNF-4* gene and its expression during mouse embryogenesis. Mech. Dev..

[25] Li J., Ning G., Duncan S.A. (2000). Mammalian hepatocyte differentiation requires the transcription factor HNF-4α. Genes Dev..

[26] Bertero A., Madrigal P., Galli A., Hubner N.C., Moreno I., Burks D., Brown S., Pedersen R.A., Gaffney D., Mendjan S. (2015). Activin/nodal signaling and NANOG orchestrate human embryonic stem cell fate decisions by controlling the H3K4me3 chromatin mark. Genes Dev..

[27] Heslop J.A., Duncan S.A. (2018). The use of human pluripotent stem cells for modelling liver development and disease. Hepatology.

[28] Basma H., Soto-Gutierrez A., Yannam G.R., Liu L., Ito R., Yamamoto T., Ellis E., Carson S.D., Sato S., Chen Y. (2009). Differentiation and transplantation of human embryonic stem cell-derived hepatocytes. Gastroenterology.

[29] Cai J., Zhao Y., Liu Y., Ye F., Song Z., Qin H., Meng S., Chen Y., Zhou R., Song X. (2007). Directed differentiation of human embryonic stem cells into functional hepatic cells. Hepatology.

[30] Si-Tayeb K., Noto F.K., Nagaoka M., Li J., Battle M.A., Duris C., North P.E., Dalton S., Duncan S.A. (2010). Highly efficient generation of human hepatocyte-like cells from induced pluripotent stem cells. Hepatology.

[31] Hay D.C., Zhao D., Fletcher J., Hewitt Z.A., McLean D., Urruticoechea-Uriguen A., Black J.R., Elcombe C., Ross J.A., Wolf R. (2008). Efficient differentiation of hepatocytes from human embryonic stem cells exhibiting markers recapitulating liver development in vivo. Stem Cells.

[32] Schwartz R.E., Linehan J.L., Painschab M.S., Hu W.S., Verfaillie C.M., Kaufman D.S. (2005). Defined conditions for development of functional hepatic cells from human embryonic stem cells. Stem Cells Dev..

[33] Touboul T., Hannan N.R., Corbineau S., Martinez A., Martinet C., Branchereau S., Mainot S., Strick-Marchand H., Pedersen R., Di Santo J. (2010). Generation of functional hepatocytes from human embryonic stem cells under chemically defined conditions that recapitulate liver development. Hepatology.

[34] Agarwal S., Holton K.L., Lanza R. (2008). Efficient differentiation of functional hepatocytes from human embryonic stem cells. Stem Cells.

[35] Delaforest A., Nagaoka M., Si-Tayeb K., Noto F.K., Konopka G., Battle M.A., Duncan S.A. (2011). HNF4A is essential for specification of hepatic progenitors from human pluripotent stem cells. Development.

[36] Wang J.C., Stafford J.M., Granner D.K. (1998). SRC-1 and GRIP1 coactivate transcription with hepatocyte nuclear factor 4. J. Biol. Chem..

[37] Qu X., Lam E., Doughman Y.Q., Chen Y., Chou Y.T., Lam M., Turakhia M., Dunwoodie S.L., Watanabe M., Xu B. (2007). Cited2, a coactivator of HNF4α, is essential for liver development. EMBO J..

[38] Dell H., Hadzopoulou C.M. (1999). CREB-binding protein is a transcriptional coactivator for hepatocyte nuclear factor-4 and enhances apolipoprotein gene expression. J. Biol. Chem..

[39] Yoshida E., Aratani S., Itou H., Miyagishi M., Takiguchi M., Osumu T., Murakami K., Fukamizu A. (1997). Functional association between CBP and HNF4 in trans-activation. Biochem. Biophys. Res. Commun..

[40] Barrero M.J., Malik S. (2006). Two functional modes of a nuclear receptor-recruited arginine methyltransferase in transcriptional activation. Mol. Cell..

[41] Malik S., Roeder R.G. (2010). The metazoan Mediator co-activator complex as an integrative hub for transcriptional regulation. Nat. Rev. Genet..

[42] Malik S., Karathanasis S.K. (1996). TFIIB-directed transcriptional activation by the orphan nuclear receptor hepatocyte nuclear factor 4. Mol. Cell Biol..

[43] Soutoglou E., Talianidis I. (2002). Coordination of PIC assembly and chromatin remodeling during differentiation-induced gene activation. Science.

[44] Si-Tayeb K., Noto F.K., Sepac A., Sedlic F., Bosnjak Z.J., Lough J.W., Duncan S.A. (2010). Generation of human induced pluripotent stem cells by simple transient transfection of plasmid DNA encoding reprogramming factors. BMC Dev. Biol..

[45] Mallanna S.K., Duncan S.A. (2013). Differentiation of hepatocytes from pluripotent stem cells. Curr. Protoc. Stem Cell Biol..

[46] Nagaoka M., Si-Tayeb K., Akaike T., Duncan S.A. (2010). Culture of human pluripotent stem cells using completely defined conditions on a recombinant E-cadherin substratum. BMC Dev. Biol..

[47] Battle M.A., Bondow B.J., Iverson M.A., Adams S.J., Jandacek R.J., Tso P., Duncan S.A. (2008). GATA4 is essential for jejunal function in mice. Gastroenterology.

[48] Yu Y., Liu H., Ikeda Y., Amiot B.P., Rinaldo P., Duncan S.A., Nyberg S.L. (2012). Hepatocyte-like cells differentiated from human induced pluripotent stem cells: Relevance to cellular therapies. Stem Cell Res..

[49] D’Amour K.A., Agulnick A.D., Eliazer S., Kelly O.G., Kroon E., Baetge E.E. (2005). Efficient differentiation of human embryonic stem cells to definitive endoderm. Nat. Biotechnol..

[50] Bailly A., Torres-Padilla M.E., Tinel A.P., Weiss M.C. (2001). An enhancer element 6 kb upstream of the mouse HNF4α1 promoter is activated by glucocorticoids and liver-enriched transcription factors. Nucleic Acids Res..

[51] Torres-Padilla M.E., Fougere-Deschatrette C., Weiss M.C. (2001). Expression of HNF4α isoforms in mouse liver development is regulated by sequential promoter usage and constitutive 3’ end splicing. Mech. Dev..

[52] Landt S.G., Marinov G.K., Kundaje A., Kheradpour P., Pauli F., Batzoglou S., Bernstein B.E., Bickel P., Brown J.B., Cayting P. (2012). ChIP-seq guidelines and practices of the ENCODE and modENCODE consortia. Genome Res..

[53] Liu T., Ortiz J.A., Taing L., Meyer C.A., Lee B., Zhang Y., Shin H., Wong S.S., Ma J., Lei Y. (2011). Cistrome: An integrative platform for transcriptional regulation studies. Genome Biol..

[54] Jiang G., Sladek F.M. (1997). The DNA binding domain of hepatocyte nuclear factor 4 mediates cooperative, specific binding to DNA and heterodimerization with the retinoid X receptor α. J. Biol. Chem..

[55] Twaroski K., Mallanna S.K., Jing R., DiFurio F., Urick A., Duncan S.A. (2015). FGF2 mediates hepatic progenitor cell formation during human pluripotent stem cell differentiation by inducing the WNT antagonist NKD1. Genes Dev..

[56] Jing R., Duncan C.B., Duncan S.A. (2017). A small-molecule screen reveals that HSP90β promotes the conversion of induced pluripotent stem cell-derived endoderm to a hepatic fate and regulates HNF4A turnover. Development.

[57] Odom D.T., Dowell R.D., Jacobsen E.S., Gordon W., Danford T.W., MacIsaac K.D., Rolfe P.A., Conboy C.M., Gifford D.K., Fraenkel E. (2007). Tissue-specific transcriptional regulation has diverged significantly between human and mouse. Nat. Genet..

[58] Fang B., Mane-Padros D., Bolotin E., Jiang T., Sladek F.M. (2012). Identification of a binding motif specific to HNF4 by comparative analysis of multiple nuclear receptors. Nucleic Acids Res..

[59] Schmidt D., Wilson M.D., Ballester B., Schwalie P.C., Brown G.D., Marshall A., Kutter C., Watt S., Martinez-Jimenez C.P., Mackay S. (2010). Five-vertebrate ChIP-seq reveals the evolutionary dynamics of transcription factor binding. Science.

